# Gestational Diabetes Mellitus Is Associated with Differences in Human Milk Hormone and Cytokine Concentrations in a Fully Breastfeeding United States Cohort

**DOI:** 10.3390/nu14030667

**Published:** 2022-02-04

**Authors:** Yuni Choi, Emily M. Nagel, Harmeet Kharoud, Kelsey E. Johnson, Tipper Gallagher, Katy Duncan, Elyse O. Kharbanda, David A. Fields, Cheryl A. Gale, Katherine Jacobs, David R. Jacobs, Ellen W. Demerath

**Affiliations:** 1 Division of Epidemiology and Community Health, University of Minnesota School of Public Health, Minneapolis, MN 55454, USA; nagel127@umn.edu (E.M.N.); kharo001@umn.edu (H.K.); jens0490@umn.edu (T.G.); jacob004@umn.edu (D.R.J.J.); ewd@umn.edu (E.W.D.); 2 Department of Genetics, Cell Biology, and Development, University of Minnesota-Twin Cities, Minneapolis, MN 55454, USA; kej@umn.edu; 3 Department of Pediatrics, University of Oklahoma College of Medicine, Oklahoma City, OK 73104, USA; Katy-Duncan@ouhsc.edu (K.D.); David-Fields@ouhsc.edu (D.A.F.); 4 HealthPartners Institute, Bloomington, MN 55425, USA; Elyse.O.Kharbanda@healthpartners.com; 5 Division of Neonatology, Department of Pediatrics, University of Minnesota Medical School, Minneapolis, MN 55454, USA; galex012@umn.edu; 6 Division of Maternal-Fetal Medicine, Department of Obstetrics, Gynecology & Women’s Health, University of Minnesota School of Medicine, Minneapolis, MN 55455, USA; gran0254@umn.edu

**Keywords:** breastmilk, human milk, hormones, cytokines, prospective cohort, gestational diabetes mellitus

## Abstract

It is unclear whether gestational diabetes mellitus (GDM) alters breast milk composition. We prospectively examined associations of GDM status with concentrations of six potentially bioactive elements (glucose, insulin, C-reactive protein (CRP), interleukin-6 (IL-6), leptin, and adiponectin) in human milk. These were measured at both 1 and 3 months postpartum in 189 fully breastfeeding women. Mixed-effects linear regression assessed GDM status-related differences in these milk bioactives, adjusting for demographics, maternal factors, and diet. At 1 and 3 months postpartum, milk CRP was higher (1.46 ± 0.31 ng/mL; *p* < 0.001 and 1.69 ± 0.31 ng/mL; *p* < 0.001) in women with GDM than in women without GDM, whereas milk glucose (−5.23 ± 2.22 mg/dL; *p* = 0.02 and −5.70 ± 2.22; *p* = 0.01) and milk insulin (−0.38 ± 0.17 μIU/mL; *p* = 0.03 and −0.53 ± 0.17; *p* = 0.003) were lower in women with GDM. These significant associations remained similar after additional adjustment for maternal weight status and its changes. No difference was found for milk IL-6, leptin, and adiponectin. There was no evidence of association between these milk bioactive compounds and 1 h non-fasting oral glucose challenge serum glucose in the women without GDM. This prospective study provides evidence that potentially bioactive elements of human milk composition are altered in women with GDM.

## 1. Introduction

Gestational diabetes mellitus (GDM) is defined as any degree of glucose intolerance that develops or manifests during pregnancy and affects about 7% of pregnancies in the US [1]. The majority of GDM cases is the result of β-cell dysfunction on a background of chronic insulin resistance throughout pregnancy, and thus both β-cell impairment and tissue insulin resistance are important components of GDM pathogenesis [2,3]. Potential risk factors for GDM include excessive weight gain, unhealthy diet (e.g., high in animal-based foods and highly-processed sugary foods), advanced maternal age, and a family history of insulin resistance and/or diabetes [2,3]. GDM is associated with subsequent health problems, including maternal cardiovascular disease and type 2 diabetes, shorter lactation duration, and short- and long-term adverse health outcomes in their children [2,3,4]. In particular, GDM increases the risk of obesity, impaired glucose tolerance, and type 2 diabetes in the child [5,6].

Exclusive breastfeeding for the first 6 months of life is recommended for all infants because it is associated with reduced risk of infant wheeze and asthma [7,8], gastrointestinal infection [9], and possibly childhood overweight and obesity [10]. However, the composition of human milk is not uniform among women, and the impact of maternal clinical factors on this variation is now being investigated [11]. It has been hypothesized that GDM may alter human milk in ways that could adversely program infant appetite, metabolic rate, and gut microbiome [12]. It is important to better understand GDM and other maternal factors as they relate to milk composition variation because of their potential to influence growth in children at high risk of future obesity, and because this information is necessary in order to design interventions that can optimize this critical first food.

The concentrations of hormones and cytokines, including insulin, leptin, adiponectin, ghrelin, and pro- and anti-inflammatory factors, were recently found to vary in human milk [11], which is influenced by maternal metabolic conditions, such as obesity [13,14,15]. Data from our group previously showed that maternal body mass index (BMI) and gestational weight gain were positively associated with milk leptin, insulin, and C-reactive protein (CRP) and inversely associated with adiponectin [16,17]. A recent study from our group has also documented that milk insulin, leptin, and glucose concentrations predicted cessation in breastfeeding exclusivity and initiation of formula use in non-GDM women [4]. Further, these milk hormones and cytokines are transported into infant circulation and act locally on the infant gut epithelium to regulate diverse systems including the developing infant microbiome and intestinal epithelium and the neuronal control of appetite and satiety [11,18]. Given that GDM is a more hyperglycemic, insulin resistant, and pro-inflammatory state than is obesity [19,20,21], we hypothesized that milk produced by women with GDM may have even higher concentrations of these factors than seen in obese women without GDM.

Associations between GDM status and milk hormones, including insulin, adiponectin, and leptin have been inconsistent in prospective cohort studies, with some studies reporting a significant association [13] while others have found no association [13,15,22,23]. These disparate findings may be partially explained by small sample sizes, varying sample collection time points, and a lack of comprehensive control for potential confounding factors. To our knowledge, few studies have evaluated the associations between GDM status and concentrations of pro-inflammatory markers in milk, including CRP and interleukin-6 (IL-6), even though immune cell function and GDM are thought to be closely linked [24].

To address these gaps in research, we compared differences in the concentrations of glucose, insulin, CRP, IL-6, leptin, and adiponectin in human milk collected at 1 and 3 months postpartum between women with and without GDM and tested whether the magnitude of any GDM effects changed from 1 to 3 months. To assess elevated blood glucose as a potential determinant of milk differences, we also tested whether serum glucose levels after the non-fasting pregnancy oral glucose challenge test (OGCT) at ~22 weeks gestation were associated with milk hormone and cytokine concentrations in the women without GDM. Finally, we assessed whether differences in milk hormone and cytokine concentrations by GDM status were explained by weight and BMI changes across pregnancy and the first three months postpartum.

## 2. Methods

### 2.1. Study Design and Participants

Study data were collected as part of the “Maternal Metabolism, Milk, and the Microbiome” (4M) and “Mothers and Infants LinKed for Healthy Growth” (MILk) studies. The 4M study recruited 35 women diagnosed with GDM at the University of Minnesota Maternal Fetal Medicine and Women’s Health Specialists Clinics between 2017 and 2019 in Minneapolis, Minnesota. The MILk study recruited 155 women without GDM from HealthPartners clinics between 2015 and 2019 as part of a related, ongoing cohort study of human milk composition. The goal of the 4M study was to investigate the relationship of maternal GDM status to her milk microbiome characteristics and hormone concentrations, and the relationship of these milk characteristics with offspring growth rate and gut microbiome development. The eligibility criteria for enrollment of the GDM women under the 4M Study were identical to those for the recruitment of non-GDM women in the MILk study and were as follows: women aged 21–45 years old; pregnant; pre-pregnancy BMI of 18.5–40.0 kg/m^2^; giving birth to a baby weighing 2500–4500 g between 37–42 weeks of gestation; and reporting an intention to fully breastfeed their singletons for at least three months. Exclusion criteria included drinking alcohol (≥ 1 drinks/wk) or ever smoking during pregnancy or lactation; having a history of or currently presenting with diabetes mellitus (type 1 or 2); having a presumed or known congenital metabolic or endocrine disease, or a congenital illness likely to interfere with the conduct of the study; and not speaking English. After delivery, women were excluded if they had a preterm birth, a low-birth-weight infant, or were not fully breastmilk feeding their infants in the entire two weeks prior to the 1-month study visit. We defined full breastfeeding as the provision of only human milk, vitamins, water, and a cumulative total of <8 ounces of formula to the infant since birth, as well as no infant formula in the two weeks prior to the study visit and milk sample collection. Study participants were asked to provide milk samples at the clinics at 1 month, with those who attended this visit being asked to return at 3 months for infant follow-up and to provide a second milk sample if they were still producing any breast milk, including if they were partially formula feeding their infant. Women in the 4M and MILk studies followed an identical protocol. Of the 190 participants enrolled, one woman had no milk constituent measures and was omitted from analysis. All remaining women had milk bioactives assayed at 1 month, but 11 women (5.8%) did not attend their 3-month visit. Adiponectin assays have not been conducted on all 3-month samples due to lack of funding; therefore 134 of the 189 participants were missing 3-month milk adiponectin. The final analytic sample included 178 (*n* = 55 for adiponectin) women who provided milk samples both at 1 and 3 months.

All participants provided written informed consent, and the institutional review boards at the University of Minnesota and Health Partners Institute approved all study protocols.

### 2.2. Ascertainment of Gestational Diabetes Mellitus

GDM status was defined on the basis of serum glucose laboratory results in the medical record. GDM was diagnosed using the following procedure: at 26 to 28 weeks of gestation, a non-fasting 1 h 50 g OGCT was administered. Serum glucose levels greater than 130 mg/dL resulted in a subsequent 3 h 100 g oral glucose tolerance test (OGTT). The diagnosis of GDM was made if at least two of the following four glucose levels were met or exceeded: 95 mg/dL (fasting), 180 mg/dL (1 h), 155 mg/dL (2 h), and 140 mg/dL (3 h) [25].

### 2.3. Assessment of Covariates

Pre-pregnancy BMI (kg/m^2^) was estimated using the first available height (m) and weight (kg) in the pregnancy medical record (no later than 8 weeks gestation). Gestational weight gain was calculated by delivery weight minus the pre-pregnancy weight. The difference between maternal delivery weight and body weight obtained at 1-month and 3-month postpartum visits was used to compute postpartum weight loss. Maternal postpartum height and weight were directly measured by study staff at the study visits. Information on maternal race/ethnicity, educational attainment, diet quality, and breastfeeding status (full, partial, or none at 3 months postpartum) were collected through self-reported questionnaires. Diet quality was assessed using the 2015 Healthy Eating Index (HEI-2015), comprised of 13 food and nutrient components that summed to a total maximum score of 100 points—a higher score indicating a healthier diet [26]. Further information abstracted from medical records included maternal age, parity, mode of delivery (Cesarean section or vaginal birth), and gestational age at delivery (weeks).

### 2.4. Human Milk Collection and Assay

The details of milk collection and the assay procedure have been reported elsewhere [16,17]. Briefly, breast milk was obtained at 1 month (±5 days) and 3 months (±10 days) postpartum. At both time points, women provided a complete breast expression sample at the study center from the right breast using a hospital-grade electric breast pump (Medela Symphony; Medela, Inc., Zug, Switzerland), two hours after breastfeeding and between 10:00 am–12:00 pm. The volume and weight of each milk sample collected were recorded. Breast milk was gently mixed and aliquoted into microcentrifuge tubes and then stored within 20 min of collection at −80 °C until analysis. At the time of analysis, tubes were thawed, gently mixed, and then centrifuged to separate the fat and aqueous phases. Skimmed milk samples were assayed for leptin, insulin, glucose, total adiponectin, CRP, and IL-6 concentrations using enzyme-linked immunosorbent assay (ELISA) kits at the laboratory of the University of Oklahoma Health Sciences Center Metabolic Research Program. The inter- and intra-assay coefficients of variation were <13% for all biological bioactives.

### 2.5. Statistical Analysis

Maternal characteristics before, during, and after pregnancy were described according to GDM status, and tested using chi-square tests for categorical data and t-tests for continuous data. All covariates were available for >95% women and were replaced with average values, and a missing indicator was added for categorical variables among non-missing values to preserve the milk constituent sample size. Missing the month 3 visit was judged to be potentially informative; therefore, we tested the frequency of month 3 visit attendance by GDM status.

We used multivariable mixed-effects linear regression to examine the associations of GDM status with the six milk bioactives (glucose, insulin, CRP, IL-6, leptin, and adiponectin) at 1 and 3 months postpartum in 178 women with complete milk composition data. Given that there were no differences in 1 month data between those who attended and those who did not attend at 3 months, we restricted the data to those who attended both visits. These models included fixed effects for time and GDM × time interaction to estimate time trend and whether GDM associations differed at 1 vs. 3 months, while taking into account the correlation across repeated observations. To normalize skewed distributions and to stabilize variance, all milk constituent values (except for glucose) were natural log transformed prior to analysis. Multivariable models additionally included maternal age, parity, self-identified ethnicity (Hispanic, non-Hispanic, or missing indicator), self-identified race (White, African-American or Black, Asian, Other, or missing indicator), education (high school/GED/associate’s, bachelor’s, or graduate degree), mode of delivery (vaginal, Cesarean, or missing indicator), maternal dietary quality (HEI) score, and gestational age of infant at birth. Pre-pregnancy BMI, gestational weight gain, and postpartum weight loss at 1 and 3 months were added in a third set of models to explore whether these adiposity measures mediated the association between GDM status and changes in milk hormones and cytokines. Next, we examined serum glucose concentration (derived from OGCT results) as a continuous variable in those without GDM using mixed-effects regression models and adjusting for the same covariates. To investigate potential bias related to non-attendance at 3 months postpartum, a sensitivity analysis of a single measure at 1 month postpartum was cross-sectionally analyzed with 154 participants. Statistical analysis was completed using SAS (version 9.4), and results with a two-sided *p* < 0.05 were considered significant.

## 3. Results

Of 189 mothers, 100% were fully breastfeeding their infants at 1 month as dictated by the inclusion and exclusion criteria, and 88% were fully breastfeeding at 3 months. Participant characteristics are shown in Table 1. Compared to women without GDM, those with GDM were older, had a younger gestational age at birth, had higher BMI before pregnancy and after delivery (at 3 months), and were more likely to self-identify as African-American/Black, Asian, or American Indian/Alaska Native and to have had a Cesarean delivery. Women with GDM also tended to have lower gestational weight gain, lower proportion of excessive weight gain (as recommended by the Institute of Medicine), and lower diet quality (as assessed by the HEI) at both 1 and 3 months postpartum. Women without GDM continued to lose weight from 1 to 3 months postpartum, but those with GDM tended to regain some weight by 3 months. BMI decreased from 1 month to 3 months among women without GDM, while it increased among those with GDM.

The characteristics at pre-pregnancy and 1 month postpartum were further stratified by study attendance status at 3 months postpartum (Supplemental Table S1) to assess whether missingness at that point was informative (associated with having GDM or its correlates). Women with GDM had lower attendance at 3 months postpartum (8/35, 22%) than those without GDM (3/154, 2%). Among women with GDM, non-attenders at 3 months had a higher educational level and were more likely to be self-identified as African-American/Black, Asian, or American Indian/Alaska Native. There were no differences in milk hormone and cytokine concentration at 1 month by the 3-month visit attendance status among women with GDM. The numerical findings for the 3 people without GDM who did not attend at 3 months are not interpretable.

Between 1 and 3 months postpartum, the unadjusted mean change in concentrations of CRP (−0.50 ± 0.97), IL-6 (−0.69 ± 1.29), leptin (−0.20 ± 0.55), and adiponectin (−0.22 ± 0.37) dropped significantly (*p* < 0.05 for each) in the overall study sample. There were no significant changes from 1 to 3 months postpartum in milk glucose (−0.79 ± 8.95) or milk insulin (−0.004 ± 0.63; *p* > 0.05 for each).

In multivariable-adjusted mixed effects models (model 1), milk CRP was higher in those with GDM at both 1 month (1.46 ± 0.31 ng/mL; *p* < 0.001) and 3 months postpartum (1.69 ± 0.31 ng/mL; *p* < 0.001) than in those without GDM. Milk glucose was lower in women with GDM than those without GDM at both 1 month (−5.23 ± 2.22; *p* = 0.02) and 3 months (−5.70 ± 2.22; *p* = 0.01), and milk insulin was also lower in the GDM group at both time points (−0.38 ± 0.17; *p* = 0.03 and −0.53 ± 0.17; *p* = 0.003, respectively) (Table 2). There was no evidence of a difference in the time trend in any of the milk analytes by GDM status (*p* for GDM × time interaction > 0.05 for all). The estimates remained similar after additional adjustment for pre-pregnancy BMI, gestational weight gain, and postpartum weight loss at each time point (model 2). Concentrations of IL-6, leptin, and adiponectin did not differ by GDM status. The results of the sensitivity analysis where we tested only 1 month milk hormone and cytokine differences cross-sectionally were similar to the primary findings (data not shown). In the analysis of OGCT glucose concentrations with milk hormone and cytokine concentrations at 1 month among women without GDM, OGCT glucose was inversely associated with 1 month milk IL-6 (*p* = 0.04; model 2 in Table 3) but was not associated with other bioactives.

## 4. Discussion

In this prospective cohort of fully breastfeeding mothers, women with GDM had higher milk CRP and lower milk glucose and insulin concentrations at both 1 and 3 months postpartum than women without GDM. The associations were independent of pre-pregnancy BMI and gestational and postpartum weight changes. Maternal OGCT blood glucose concentrations did not predict milk hormone and cytokine concentrations in non-GDM mothers.

The clinical implications of human milk composition is of increasing interest, particularly in regard to enhancing growth and development and the gut microbiome in infancy, as well as reducing maternal and infant adverse health outcomes [2,3,18,27,28]. Although GDM is one of the most common clinical conditions affecting pregnant women in the US and has known associations with lactation dysfunction, as well as adverse effects on both maternal and infant health, epidemiological evidence on the association between GDM status and milk composition is scarce [28]. Most existing studies are small, examined few milk components, and varied in their control for potential confounding factors and technical sources of variation (time of day, stage of lactation). Together, these characteristics may have contributed to inconsistent results across studies [28].

CRP is a homopentameric protein that is primarily synthesized in hepatocytes and is a marker for systemic low-grade inflammation [29]. Interestingly, in lactating women, milk CRP concentration is higher than in blood, suggesting upregulated expression of this protein by the lactocyte [30]. The higher milk CRP concentration in women with GDM reported in this study is novel and extends a previous report by our group showing a strong positive association of milk CRP with maternal BMI and gestational weight gain [16]. Comparing to those findings, milk CRP in women with GDM was higher than those without GDM, independent of maternal adiposity measures as assessed before, during, and after pregnancy. There has been no direct mechanistic investigation of how milk CRP levels are increased in women with GDM, but there is considerable evidence that the maternal immune system is altered by the metabolic dysregulation of GDM. Diabetes is a condition associated with chronic low-grade inflammation characterized by elevated infiltration and activation of innate and adaptive immune cells and eventually induces the development of adipose tissue (including mammary adipose tissue) inflammation, insulin resistance, and endothelial dysfunction [31,32]. Dysregulation of adipose tissue results in the production of pro-inflammatory cytokines such as tumor necrosis factor alpha (TNF-α) and interleukin-6 (IL-6), which is related to increased circulating CRP, while lowering the synthesis of anti-inflammatory adipokines such as adiponectin [33,34]. GDM specifically is associated with increases in serum levels TNF-α [35] and with increased expression of genes such as NOS2 and SFTPD in leukocytes [24]. Further, TNF-α, IL-1, and Cox-2 levels were increased, as were aromatase mRNA and activity levels in the mammary gland and visceral fat of obese mice [36]. Due to the lack of postpartum glucose data in our study, we were unable to assess the association between postpartum glucose intolerance and milk CRP. However, given that GDM is associated with an increased risk of recurring GDM and both a short-term and long-term risk of maternal metabolic syndrome, type 2 diabetes, and cardiovascular disease [2,3,37], we know that endocrine dysfunction and corresponding systemic inflammation may be persistent and thereby influence milk CRP and other milk bioactives in the postpartum period.

It has been suggested that insulin concentration in breast milk may be similar to or higher than in maternal blood, and an active mechanism is presumed to drive its transport into milk by mammary epithelial cells [38]. In regard to changes to milk insulin concentration over the course of lactation, some studies, including the current cohort, did not report significant differences, whereas others found decreases over time [13,15,39]. In our past work in non-diabetic women, we reported that elevated milk insulin concentrations from 1 to 3 months were associated with more frequent formula initiation [4]. Several studies also examined the association of diabetes diagnostic markers or GDM with milk insulin, but these results were inconsistent across studies and currently provide limited evidence [13,15,22,23,38,39]. Ley et al. reported a positive association between maternal serum fasting glucose and HOMA-IR and insulin in mature milk and no association between GDM and milk insulin [15]. Two later studies found that maternal fasting serum insulin, glucose, HOMA-IR, and GDM were all positively related to milk insulin concentration [13,39]. In contrast, milk insulin concentration was observed to be lower in women with a history of type 1 or 2 diabetes compared with a control group [38]. We likewise found that milk insulin concentration was lower in women with GDM. Other studies found no association between either GDM or a combined diabetes definition (type 1 or 2 diabetes with GDM) and milk insulin concentration [22,23]. The widely varying results between studies may be explained by variations in type of diabetes studied (a history of type 1 or 2 or GDM), analytical comparison groups (some did not compare GDM vs. healthy groups), the time of milk sample collection, method of milk collection, sample sizes, and the characteristics of study sample (racial difference and clinical conditions). It is likely that the lack of a clear signal in the case of milk insulin also stems from the highly varied metabolic status in the postpartum period across different women with GDM. An HbA1c test at milk study collection would assist in discriminating between women with glucose dysregulation only in pregnancy and those for whom it is persisting during lactation, thereby controlling for some of this inter-individual variation.

One of the interesting findings in our study was that milk glucose was lower in women with GDM than in those without GDM. In contrast, Kaushik et al. found no difference in milk glucose concentration associated with GDM [40], while Whitmore et al. showed that milk glucose concentration was higher in women with a history of type 1 or 2 diabetes compared to controls (they did not examine women with GDM) [38]. We also found that OGCT glucose did not predict milk glucose in women without GDM. In the absence of blood sugar control after diagnosis, blood glucose is likely to be higher and more variable in women with GDM. Our initial expectation was that the concentration of glucose and insulin in the mother’s blood would be proportional to the concentration of glucose and insulin in breast milk, based on the equilibrium resulting from chemical mass action [41]. However, our findings are that women with GDM had lower milk glucose levels, implying that milk glucose is regulated, at least partially, independent of blood glucose. High glucose concentration in the blood is the result of loss of homeostatic control. We speculated that the same loss of homeostasis may act in an inverse way on the transport of glucose from the mother’s blood to the milk. Glucose is a precursor for lactose, the primary sugar in human milk, and consequently, the mammary gland requires an adequate supply of glucose to produce milk [42]. While some glucose is taken up for the production of lactose, another portion is secreted from the mammary epithelial cells into the milk via apical transporters [43]. Neville et al. found that milk glucose concentrations were proportional to the rate of milk secretion, such that glucose concentrations in the mammary cells decreased as milk synthesis decreased [44]. Thus, glucose concentration in human milk is highly controlled given its likely importance in milk secretion, and may be altered by GDM through mammary cell dysregulation in a way that does not correlate with maternal serum concentration. This interesting finding is novel and merits further investigation.

We hypothesized that bioactives that are different by GDM status group would also have a corresponding linear association with the OGCT glucose level in women without GDM, but this was not observed. The only bioactive associated with the 1 h OGCT glucose concentration was IL-6, but the level of significance was inconsistent across levels of adjustment and models. This association was the only one of these exploratory analyses to reach nominal statistical significance; given multiple comparisons it might have occurred by chance. Therefore, overall, we did not find evidence that variation in blood glucose response to pregnancy oral glucose challenge within the normal range is related to a variation in these milk bioactive concentrations. An examination of maternal HbA1C or fasting glucose during pregnancy and the postpartum period would be beneficial in determining whether the differences in bioactive concentrations we observed between women with and without GDM stem from glucose dysregulation specifically or from other physiological changes in women with this condition.

While we found associations between concentrations of milk CRP, glucose, and insulin and GDM status, we did not observe differences in milk IL-6, adiponectin, or leptin between women with and without GDM.

Regulation and development of mammary gland function are mediated by IL-6 and other cytokines [45,46], and breast milk is known to contain pro-inflammatory cytokines, which may have anti-inflammatory and anti-infection activities for the infant gut, with decreasing concentration over the course of lactation [47,48,49]. Few data exist on the association between GDM status and milk IL-6. We found no relationship between milk IL-6 and GDM status. This null result may be due to the low levels of IL-6 receptors in both colostrum and mature milk, as well as their low affinity [50,51]. Additionally, despite a strong link between CRP levels in blood and breast milk as observed in canine mastitis research [52], maternal serum IL-6 levels were not associated with IL-6 levels in human breast milk [53]. Studies to date have furthermore not shown a relationship of maternal adiposity to mature milk IL-6 concentrations [54]. In addition, we saw no correlation between milk CRP and milk IL-6 at 1 or 3 months. It is possible that the elevated milk CRP, but not IL-6, in women with GDM reflects the general systemic pro-inflammatory state in women with GDM and/or that the mammary epithelium is controlling passage of some but not other cytokines into human milk.

Adiponectin, a protein secreted by adipocytes, is found in high concentrations in blood and exhibits insulin-sensitizing, anti-inflammatory, and cardioprotective activities [55]. Its concentrations in blood and breast milk have a modest positive association [15], and we have previously reported that milk adiponectin is slightly lowered in maternal obesity [17]. Consistent with our observations, earlier studies reported decreases in milk adiponectin concentrations over the course of lactation [13,15]. With regard to GDM status, two previous studies and the current study found no significant difference [15,23], while one reported inverse associations of GDM and maternal serum glucose with milk adiponectin [13]. The direction of this association is consist with blood adiponectin observed in diabetic patients [56].

Leptin is an adipokine hormone generated by adipocytes that has a positive correlation with body weight, suppresses appetite, and increases energy expenditure [57]. Serum leptin was positively correlated with milk leptin, and higher concentrations in blood than in milk were observed [39,58], with a decrease in concentration over time in mature milk [13] as we have shown here as well. There is as yet little understanding of how leptin directly affects the mammary gland during lactation. The leptin receptor is expressed in the basal layer of the mammary epithelium in mice [59], but its transcripts have not been identified in mammary epithelial cells in the lumen [60], suggesting that leptin functions in the development and/or function of basally-located mammary stem cells and myoepithelial cells, rather than in milk secretion. Prior investigations found no difference in milk leptin by GDM status [13,22,23], which is consistent with our results. This is perhaps surprising given the elevation of serum leptin in women with GDM [35] and the known positive association of both serum and milk leptin with maternal adiposity [17], which was elevated in the women with GDM in this and other studies [13,23]. A greater understanding of the role of adipokines in mammary cell function and a more detailed assessment of their transport into milk are needed to explain why serum elevation of these adipokines in diabetes and obesity results in increased milk levels for some adipokines, but not others.

The strengths of our study include its prospective design, standardized sample collection protocol including milk collection timing, repeated measurements of milk bioactives, and assessment of a wide range of covariates. In addition, as one of the few studies exploring such associations, our results offer a valuable and useful perspective on how glucose tolerance alters milk bioactives. However, the following limitations should be taken into consideration when interpreting the findings of our study. The study is observational and cannot assess the causal relationship of GDM to milk composition. Our sample was largely comprised of white women, which may limit the generalizability of our results to a broader population of women. However, the biological and physiological influence of GDM on milk bioactives is unlikely to differ by race and ethnicity. In addition, the concentration of these potentially bioactive factors in milk was not measured in maternal serum, which reduces both our ability to understand the mechanisms for their appearance in milk and to assess the potential for clinical interventions among women with GDM to result in changes to their milk.

## 5. Conclusions

In summary, we found that women with GDM had higher CRP, lower insulin, and lower glucose concentrations in their breast milk than those who did not at both 1 and 3 months postpartum. The current study adds to our knowledge that GDM may play a role in the variation in milk hormone and cytokine levels. Future studies would benefit from investigating a more comprehensive set of milk compositional elements in women with GDM and should test whether alterations of milk composition in women with GDM modify the effect of breastfeeding on obesity and diabetes risk in their children.

## Figures and Tables

**Table 1:** Demographic and clinical characteristics at baseline and 1 month to 3 months postpartum according to GDM status, *n* = 189.

Variables	No GDM (*n* = 154)	GDM (*n* = 35)	*p*-Value ^a^
Age (years)	31.2 ± 4.1	34.2 ± 4.3	<0.001
Ethnicity, *n* (%)			
Hispanic or Latino	2 (1.3)	1 (2.9)	0.52
Not Hispanic or Latino	148 (98.7)	34 (97.1)	
Race, *n* (%)			
White	127 (84.1)	24 (68.6)	0.006
African American or Black	10 (6.6)	2 (5.7)	
Asian	4 (2.7)	7 (20.0)	
American Indian/Alaska Native	3 (2.0)	1 (2.9)	
Other	4 (2.7)	1 (2.9)	
Mixed race	3 (2.0)	0 (0)	
Education, *n* (%)			
High school/GED/associate’s	30 (19.5)	8 (22.9)	0.72
Bachelor’s degree	60 (39.0)	15 (42.9)	
Graduate degree	64 (41.6)	12 (34.3)	
Baseline parity, *n* (%)			
None	71 (47.0)	12 (36.4)	0.18
1	52 (34.4)	17 (51.5)	
≥2	28 (18.5)	4 (12.1)	
Gestational age at birth (years)	39.8 ± 1.1	38.2 ± 1.9	<0.001
Mode of delivery, *n* (%)			<0.001
Vaginal	120 (83.3)	16 (48.5)	
Cesarean	24 (16.7)	17 (51.5)	
Pre-pregnancy BMI (kg/m^2^)	26.5 ± 4.7	29.6 ± 7.4	0.002
1 h 50 g OGCT result (mg/dL)	107 ± 17.7	158 ± 19.4	<0.001
Gestational weight gain of mother (kg)	13.6 ± 6	9.8 ± 5.3	0.001
Excessive gestational weight gain (IOM) yes, *n* (%) ^b^	77 (52.0)	11 (32.4)	0.03
Breast milk volume (mL)			
1 month postpartum	72.9 ± 43.2	71.6 ± 45.3	0.87
3 months postpartum	84.3 ± 51.5	86.1 ± 48.9	0.86
Exclusive breastfeeding at 3 months, *n* (%)	143 (94.1)	23 (92.0)	0.69
Postpartum weight loss (kg)			
1 month postpartum	9.9 ± 4.1	10.3 ± 2.3	0.57
3 months postpartum	10.9 ± 4.8	9.7 ± 3.5	0.21
Postpartum BMI (kg/m^2^)			
1 month postpartum	27.9 ± 4.4	29.5 ± 6.0	0.07
3 months postpartum	27.3 ± 4.6	29.9 ± 6.2	0.02
Diet quality score assessed via HEI			
1 month postpartum	65.8 ± 8.4	62.1 ± 10.6	0.04
3 months postpartum	66.5 ± 8.9	63.0 ± 7.0	0.09

BMI, body mass index; GDM, gestational diabetes mellitus; GED, graduate equivalency degree; HEI, healthy eating index; OGCT, oral glucose challenge test. Values are reported as the mean± SD, unless noted as percentage. No. of missing is as follows: maternal age, N=2; ethnicity, N=4; race, N=3; parity, N=5; gestational age at birth, N=3; mode of delivery, N=12; pre-pregnancy BMI, N=2;1 h 50 g OGCT result, N=7; gestational weight gain, N=6; breast milk volume at 1 month, N=1 and at 3 months, N=1; exclusive breastfeeding at 3 months, N=12; postpartum weight loss at 1 month, N=6 and at 3 months, N=5; postpartum BMI at 1 month, N=3 and 3 months, N=5; HEI at 1 month, N=10 and at 3 months, N=10. ^a^ Evaluated with chi-square tests for categorical variables and t-test for continuous variables. ^b^ Beyond the IOM weight gain guidelines for pregnancy was considered excessive weight gain. The recommended ranges by pre-pregnancy weight status are the following: 12.5−18 kg for underweight (BMI <18.5 kg/m^2^); 11.5–16 kg for normal weight (BMI 18.5−24.9 kg/m^2^); 7−11.5 kg for overweight (BMI 25−29.9 kg/m^2^); and 5–9 kg for obese (BMI ≥30 kg/m^2^).

**Table 2:** GDM-related differences in human milk hormone and cytokine concentrations at 1 and 3 months postpartum in 178 women with complete milk composition data ^a^.

	Mean Concentrations				Regression Estimates (Concentration in GDM as Compared to Non-GDM)					
	Non-GDM		GDM		Unadjusted Model		Multivariable		Multivariable	
							Model 1 ^c^		Model 2 ^d^	
	*n*	Unadjusted Mean ± SE ^b^	*n*	Unadjusted Mean ± SE ^b^	*ß ± SE*	*p*-Value	*ß ± SE*	*p*-Value	*ß ± SE*	*p*-Value
Milk glucose, mg/dL										
1 month postpartum	151	29.66 ± 0.76	27	26.98 ± 1.79	−2.68 ± 1.94	0.17	−5.23 ± 2.22	0.02	−4.77 ± 2.24	0.03
3 months postpartum	151	28.94 ± 0.76	27	25.82 ± 1.79	−3.11 ± 1.94	0.11	−5.70 ± 2.22	0.01	−5.45 ± 2.23	0.02
Log milk insulin, μIU/mL										
1 month postpartum	151	3.17 ± 0.06	27	2.91 ± 0.14	−0.26 ± 0.15	0.10	−0.38 ± 0.17	0.03	−0.45 ± 0.17	0.007
3 months postpartum	151	3.18 ± 0.06	27	2.78 ± 0.14	−0.40 ± 0.15	0.01	−0.53 ± 0.17	0.003	−0.60 ± 0.16	<0.001
Log milk CRP, ng/mL										
1 month postpartum	151	4.38 ± 0.10	27	5.89 ± 0.24	1.50 ± 0.26	<0.001	1.46 ± 0.31	<0.001	1.38 ± 0.29	<0.001
3 months postpartum	151	3.85 ± 0.10	27	5.58 ± 0.24	1.74 ± 0.26	<0.001	1.69 ± 0.31	<0.001	1.58 ± 0.29	<0.001
Log milk IL-6, pg/mL										
1 month postpartum	151	1.66 ± 0.11	27	1.54 ± 0.26	−0.12 ± 0.28	0.67	0.003 ± 0.33	0.99	−0.16 ± 0.32	0.60
3 months postpartum	151	1.00 ± 0.11	27	0.71 ± 0.26	−0.29 ± 0.28	0.31	−0.17 ± 0.33	0.60	−0.23 ± 0.32	0.47
Log milk leptin, pg/mL										
1 month postpartum	151	6.23 ± 0.07	27	6.44 ± 0.16	0.21 ± 0.17	0.22	0.04 ± 0.19	0.85	0.05 ± 0.14	0.74
3 months postpartum	151	6.03 ± 0.07	27	6.19 ± 0.16	0.16 ± 0.17	0.36	−0.01 ± 0.19	0.96	−0.12 ± 0.14	0.41
Log milk adiponectin, ng/mL										
1 month postpartum	28	2.99 ± 0.03	27	2.90 ± 0.08	−0.09 ± 0.08	0.27	−0.07 ± 0.10	0.44	−0.05 ± 0.10	0.61
3 months postpartum	28	2.73 ± 0.06	27	2.65 ± 0.08	−0.07 ± 0.10	0.47	−0.06 ± 0.11	0.61	−0.05 ± 0.11	0.67

CRP, C-reactive protein; GDM, gestational diabetes mellitus; IL-6, interleukin-6. ^a^ Separate mixed-effects linear regression models with GDM x month interaction were used to test the association of GDM vs. non-GDM and each repeated human milk constituent concentrations, assuming GDM and time as fixed factors when applicable. This model included participants attending both visits at month 1 and 3 months. GDM × time interaction was not significant (*p* > 0.05). ^b^ Least squares means computed from the unadjusted linear regression models. ^c^ Model was adjusted for maternal age, parity, self-identified ethnicity (Hispanic, non-Hispanic, or missing indicator), self-identified race (White, African American or Black, Asian, Other, or missing indicator), education (high school/GED/associate’s, bachelor’s degree, or graduate degree), mode of delivery (vaginal, Cesarean, or missing indicator), HEI, and infant’s gestational age at birth. ^d^ Additionally adjusted for pre-pregnancy BMI, gestational weight gain, and postpartum weight loss at 1 and 3 months.

**Table 3:** Association of OGCT result at 1 h and human milk hormone and cytokine concentrations at 1 month in women without GDM ^a^.

	**Continuous (per 1-SD) among Non-GDM**	
	**(Median 108 mg/dL), *n* = 149**	
	***ß ± SE***	***p*-Value**
Milk glucose, mg/dL		
Unadjusted model	0.82 ± 0.78	0.30
Multivariable model 1 ^b^	1.19 ± 0.84	0.16
Multivariable model 2 ^c^	1.43 ± 0.87	0.10
Log milk insulin, μIU/mL		
Unadjusted model	0.02 ± 0.05	0.69
Multivariable model 1 ^b^	0.03 ± 0.05	0.55
Multivariable model 2 ^c^	0.02 ± 0.05	0.74
Log milk CRP, ng/mL		
Unadjusted model	0.22 ± 0.10	0.03
Multivariable model 1 ^b^	0.21 ± 0.11	0.06
Multivariable model 2 ^c^	0.16 ± 0.10	0.13
Log milk IL-6, pg/mL		
Unadjusted model	−0.11 ± 0.11	0.32
Multivariable model 1 ^b^	−0.12 ± 0.12	0.31
Multivariable model 2 ^c^	−0.23 ± 0.11	0.04
Log milk leptin, pg/mL		
Unadjusted model	0.10 ± 0.06	0.09
Multivariable model 1 ^b^	0.10 ± 0.06	0.11
Multivariable model 2 ^c^	0.08 ± 0.04	0.07
Log milk adiponectin, ng/mL		
Unadjusted model	−0.05 ± 0.03	0.12
Multivariable model 1 ^b^	−0.03 ± 0.03	0.28
Multivariable model 2 ^c^	−0.03 ± 0.03	0.42

CRP, C-reactive protein; IL-6, interleukin-6. OGCT, oral glucose challenge test. ^a^ Separate linear regression models were used to evaluate the relationship between the OGCT outcome as continuous (1-SD higher 17.7 mg/dL) and human milk constituent concentration at 1 month. ^b^ Model was adjusted for maternal age, parity, self-identified ethnicity (Hispanic, non-Hispanic, or missing indicator), self-identified race (White, African American or Black, Asian, Other, or missing indicator), education (high school/GED/associate’s, bachelor’s degree, or graduate degree), mode of delivery (vaginal, Cesarean, or missing indicator), HEI, and infant’s gestational age at birth. ^c^ Additionally adjusted for pre-pregnancy BMI, gestational weight gain, and postpartum weight loss at 1 and 3 months.

## Data Availability

The data underlying this article are available from the MILk cohort team upon reasonable request.

## Supplementary Materials

The following are available online: https://www.mdpi.com/article/10.3390/nu14030667/s1. Table S1. Demographic, clinical characteristics, and human milk bioactive concentrations at baseline and 1 month postpartum according to GDM status, stratified by attendance status at 3 months postpartum.
