# Supplementary material for: Gestational Diabetes Mellitus Is Associated with Differences in Human Milk Hormone and Cytokine Concentrations in a Fully Breastfeeding United States Cohort
Source: Nutrients. 2022 Feb 4;14(3):667. doi: 10.3390/nu14030667 (PMC8838140; doi:10.3390/nu14030667)
Supplement: Supplementary file 1 [file nutrients-14-00667-s001.zip › nutrients-1564985-supplementary.pdf]

SUPPLEMENTARY DATA

Table S1. Demographic, clinical characteristics, and human milk bioactive concentrations at baseline and 1 month postpartum according to GDM status, stratified by attendance status at 3 months postpartum, N=189

Variables	No GDM (n=154)		P-value [a]	GDM (n=35)		P-value [a]
	No missing month 3 (n=151)	Missing month 3 (n=3)		No missing month 3 (n=27)	Missing month 3 (n=8)	
Age	31.3±4.1	27.3±2.1	0.10	34.0±4.5	35.1±3.9	0.53
Ethnicity, n (%)						
Hispanic or Latino	2 (1.3)	0 (0)	**0.003**	1 (3.7)	0 (0)	0.58
Not Hispanic or Latino	146 (96.7)	2 (66.7)		26 (96.3)	8 (100)	
Don't know/Not available	3 (2)	1 (33.3)		–	–	
Race, n (%)						
White	127 (84.1)	0 (0)	**<0.001**	23 (85.2)	1 (12.5)	**<0.001**
African American or Black	8 (5.3)	2 (66.7)		1 (3.7)	1 (12.5)	
Asian	4 (2.7)	0 (0)		1 (3.7)	6 (75.0)	
American Indian/Alaska Native	3 (2.0)	0 (0)		1 (3.7)	0 (0)	
Other	4 (2.7)	0 (0)		1 (3.7)	0 (0)	
Mixed race	3 (2.0)	0 (0)		–	–	
Don't know/Not available	2 (1.3)	1 (33.3)		–	–	
Education, %						
High school/GED/associate's	28 (18.5)	2 (66.7)	0.09	6 (22.2)	2 (25.0)	**0.009**
Bachelor's degree	59 (39.1)	1 (33.3)		15 (55.6)	0 (0)	
Graduate degree	64 (42.4)	0 (0)		6 (22.2)	6 (75.0)	
Baseline parity, %						
None	71 (47.7)	0 (0)	0.34	7 (26.9)	5 (71.4)	0.07
1	51 (34.2)	1 (50.0)		16 (61.5)	1 (14.3)	
≥2	27 (18.1)	1 (50.0)		3 (11.5)	1 (14.3)	
Gestational age at birth	39.8±1.1	39.6±0.1	0.73	38.1±2.1	38.6±1.3	0.54
Mode of delivery, %						
Vaginal	118 (83.1)	2 (100)	0.52	14 (53.9)	2 (28.6)	0.24
Cesarean	24 (16.9)	0 (0)		12 (46.2)	5 (71.4)	
Pre-pregnancy BMI, kgm/m^2	26.4±4.6	32.8±3.7	**0.02**	29.4±7.3	30.5±8.1	0.72

1-h 50g OGCT result (mg/dL)	107.1±17.7	103±24	0.75	157.9±21.3	158.0±13.4	1.00
Gestational weight gain of mother, kg	13.6±5.9	18.2±14.8	0.28	10.1±5.7	8.8±4.0	0.55
Excessive gestational weight gain (IOM) yes, n (%)	76 (52.1)	1 (50.0)	0.76	10 (37.0)	1 (14.3)	0.26
Breast milk volume at month 1, mL	73.9±43.1	24.4±8.8	**0.049**	78.8±47.9	48.1±25.7	0.09
Postpartum weight loss at month 1, kg	10.0±4.0	3.8±11.2	**0.03**	10.4±2.4	10.1±2.0	0.79
Postpartum BMI, kg/m^2	27.7±4.3	37.4±3.9	**<0.001**	29.6±6.0	29.1±6.2	0.83
Diet quality score assessed via HEI at month 1	65.7±8.4	68.3±3.8	0.67	61.8±9.9	63.6±14.6	0.74
Milk glucose, mg/dL	29.7±9.5	18.3±9.9	**0.04**	27.0±11.7	23.7±12.5	0.49
Log milk insulin, µIU/mL	3.2±0.7	3.7±0.8	0.15	2.9±0.9	3.2±0.8	0.37
Log milk C-reactive protein, ng/mL	4.4±1.2	4.8±0.7	0.60	5.9±1.5	5.9±1.1	0.93
Log milk Interleukin-6, pg/mL	1.7±1.4	3.3±1.7	**0.04**	1.5±1.7	2.4±1.8	0.20
Log milk leptin, pg/mL	6.2±0.7	8.1±0.9	**<0.001**	6.4±0.9	6.2±1.4	0.62
Log milk adiponectin, ng/mL	3.0±0.4	4.2±1.0	**<0.001**	2.9±0.37	3.0±0.5	0.35

GDM, gestational diabetes mellitus; BMI, body mass index; HEI, healthy eating index

Values are reported as the mean± SD, unless noted as percentage. No. of missing is as follows: maternal age, N=2; ethnicity, N=4; race, N=3; parity, N=5; gestational age at birth, N=3; mode of delivery, N=12; pre-pregnancy BMI, N=2;1 h 50 g OGCT result, N=7; gestational weight gain, N=6; breast milk volume at 1 month, N=1 and at 3 months, N=1; exclusive breastfeeding at 3 months, N=12; postpartum weight loss at 1 month, N=6 and at 3 months, N=5; postpartum BMI at 1 month, N=3 and 3 months, N=5; HEI at 1 month, N=10 and at 3 months, N=10.

[a] Evaluated with chi-square tests for categorical variables and t-test for continuous variables.
